# Association of Intestinal Helminthiasis with Disseminated Leishmaniasis, Brazil

**DOI:** 10.3201/eid3009.240419

**Published:** 2024-09

**Authors:** Brady Page, Alexsandro Lago, Edgar M. Carvalho

**Affiliations:** University of California–San Diego, La Jolla, California, USA (B. Page);; Scripps Research Institute, La Jolla (B. Page);; Universidade Federal da Bahia, Salvador, Brazil (A. Lago, E.M. Carvalho);; Instituto Nacional de Ciências e Tecnologia para Doenças Tropicais, Brasília, Brazil (E.M. Carvalho);; Fundação Oswaldo Cruz, Salvador (E.M. Carvalho)

**Keywords:** helminths, leishmaniasis, *Leishmania braziliensis*, co-infection, emerging diseases, zoonoses, Brazil, parasites

## Abstract

Disseminated leishmaniasis is an emerging clinical form of *Leishmania braziliensis* infection. Evidence shows that co-infection by *L. braziliensis* and intestinal helminths does not affect clinical manifestations or response to therapy in cutaneous leishmaniasis patients. We evaluated whether co-infection was associated with those aspects in disseminated leishmaniasis patients in Brazil.

American tegumentary leishmaniasis represents a group of neglected tropical diseases caused by protozoans of the genus *Leishmania* and transmitted to humans by phlebotomine sand flies. Brazil has one of the highest incidences of leishmaniasis in the world, where the predominating species is *Leishmania braziliensis* (*1*). The disease classically manifests as the localized ulcers of cutaneous leishmaniasis (CL), but in some instances, amastigotes metastasize from the site of inoculation and lead to disseminated leishmaniasis (DL), a severe and poorly understood form of disease characterized by the presence of up to several thousand skin lesions on multiple areas of the body; DL incidence has increased severalfold in recent decades (*1*).

No histopathologic or immunologic differences have been identified between CL and DL, but several host and parasite factors have been associated with the development of DL, including *L. braziliensis* strain and immune effector cell function (*2*,*3*). Although infection with intestinal helminths has been shown to modulate host immune response to bacterial and viral infections by inducing regulatory and Th2-type T cells, co-infection with *L. braziliensis* does not definitively affect clinical or therapeutic aspects of CL (*4*–*7*). However, a previous study showed an association between helminth infection and mucocutaneous leishmaniasis (*8*). To better clarify whether the presence of helminths contributes to *L. braziliensis* dissemination, we evaluated the influence of intestinal helminthiasis on clinical manifestations and response to therapy in DL patients in Brazil.

We recruited participants during January–December 2017 at a dedicated leishmaniasis center in the endemic region of Corte da Pedra in Bahia state, Brazil. Persons 5–70 years of age with skin lesions that had been present <60 days were eligible for enrollment. Criteria for diagnosis of CL were the presence of 1–9 well-demarcated cutaneous ulcers and detection by PCR of *L. braziliensis* DNA in a punch biopsy taken from a lesion. DL was defined as the presence of >10 cutaneous lesions located on >2 noncontiguous body parts and a positive PCR. At enrollment, participants provided a stool sample. We determined the presence and quantification of intestinal helminth infection by the Kato–Katz method (*9*). All participants, regardless of enrollment status, were clinically evaluated and treated with 20 mg/kg/day of intravenous meglumine antimoniate for 20 days.

Initial clinical examination consisted of an evaluation of the size and number of cutaneous lesions. Upon 60-day and 90-day follow-up, we evaluated participants for the appearance of new lesions and response to treatment of existing lesions. We considered participants to be cured on the basis of the presence of complete re-epithelialization without elevated borders of all CL or DL lesions within 90 days after the initiation of antimonial treatment.

We enrolled a total of 99 persons with CL and 20 with DL. The median age of participants was 26 years (range 13–69 years); 76.5% of participants were male and 23.5% female (Table). Persons with DL were significantly older than patients with CL (42 vs. 24 years; p = 0.02), although we observed no correlation between age and time to cure in DL patients (R^2^ 0.06; p = 0.5) (Appendix Figure 1). Among persons with CL, age was correlated with time to cure but does not explain much of its variability (R^2^ 0.07; p = 0.04).

**Table:** Comparison of characteristics of patients with cutaneous and disseminated leishmaniasis, with and without intestinal helminthiasis, Brazil, 2017

Characteristic	All patients				Patients with disseminated leishmaniasis		
	Cutaneous leishmaniasis	Disseminated leishmaniasis	p value		With intestinal helminthiasis	Without intestinal helminthiasis	p value
Total no. patients	99	30			8	12	
Median age, y (range)	24 (13–56)	42 (18–69)	0.02*		37.5 (24–65)	49.5 (18–69)	0.6*
Sex, no. (%)							
M	76 (76.8)	15 (75)	1†		6 (75)	8 (67)	1†
F	23 (23.4)	5 (25)			2 (25)	4 (33)	
Median duration of illness, d (range)	40 (20–60)	30 (15–90)	0.18*		40 (20–90)	30 (15–90)	0.43*
Median no. lesions (range)	1 (1–8)	35.5 (11–1,500)	<0.05*		46 (11–1,500)	27 (12–102)	0.8*
Median area of largest lesion, mm^2^ (range)	224 (15–3,016)	260.5 (16–16,160)	0.3*		240 (36–16,160)	260.5 (16–1,280)	0.57*
Helminths present, % (no.)	40 (40/99)	40 (8/20)	1†				
Median no. helminth ova present on fecal microscopy (range)	6 (1–4,000)	60 (1–288)	0.08*				
Cure rate at 90 d, % (no. patients)	65.9 (60/91)	30 (6/20)	0.003*		25 (2/8)	33 (4/12)	0.78*
Median time to cure, d (range)	65 (17–390)	154.5 (45–800)	<0.001*		184.5 (60–800)	140 (45–540)	0.46*

*Significance assessed by Mann-Whitney U test. †Significance assessed by χ^2^ test.

The prevalence of intestinal helminthiasis was 40.3% (48/119 patients); we observed no difference in prevalence between the CL and DL patients (Appendix Table). The most commonly identified organisms were *Necator americanus* (23/119 patients), *Trichuris trichiuris* (19/119 patients), and *Ascaris lumbricoides* (14/119 patients). The cure rate after 90 days of treatment was significantly lower in persons with DL compared with those with CL (30% vs. 65.9%; p = 0.003), and the median time to cure was significantly longer (154.5 vs. 65 days; p<0.001) (Figure). We observed no significant difference in the median number of lesions, median area of largest lesion, cure rate at 90 days, or median time to cure between DL patients with or without intestinal helminthiasis. We also observed no correlation between quantity of helminth ova in stool and time to cure in either group (R^2^ 0.01) (Appendix Figure 2).

**Figure:**
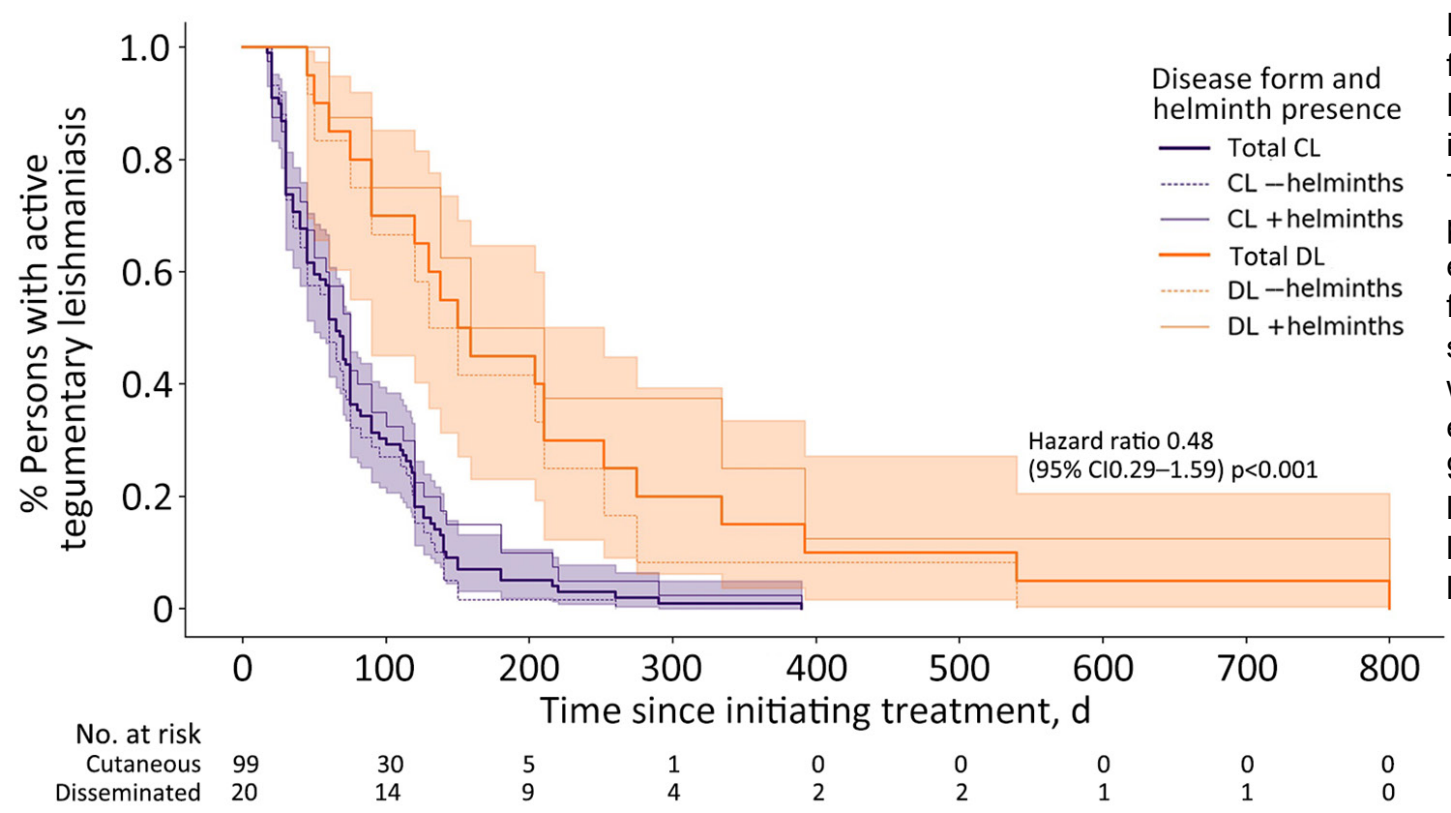
Kaplan-Meier estimates for clinical response of CL and DL stratified by the presence of intestinal helminths, Brazil, 2017. Thin dashed lines represent persons with stool parasitologic examinations that were negative for intestinal helminths; thin solid lines represent persons with positive stool parasitologic examinations; shading indicates 95% CIs. p value calculated with log-rank test. CL, cutaneous leishmaniasis; DL, disseminated leishmaniasis.

Future investigation is needed to expound upon the immunologic and strain-specific parasitologic factors that may compromise the host Th1 immune response. A decrease in Th1-associated interferon-γ and tumor necrosis factor α production may precipitate the development of DL in a minority of patients.

The role of concomitant intestinal helminth infection in the clinical aspects and therapeutic response of CL and DL has thus far remained uncertain. We found a high prevalence of intestinal helminthiasis, but we observed no difference between the CL and DL groups and no effect on outcomes of either disease form. Persons with DL were significantly older than persons with CL, although there was no significant effect within age groups on therapeutic response. Cure rates among persons with DL treated with antimonials were characteristically low compared with persons with CL (*10*).

Appendix Additional information about association of intestinal helminthiasis with disseminated leishmaniasis, Brazil.
