# Supplementary material for: Association of Intestinal Helminthiasis with Disseminated Leishmaniasis, Brazil
Source: Emerg Infect Dis. 2024 Sep;30(9):1970–2. doi: 10.3201/eid3009.240419 (PMC11347011; doi:10.3201/eid3009.240419)
Supplement: Appendix — Additional information about association of intestinal helminthiasis with disseminated leishmaniasis, Brazil. [file 24-0419-Techapp-s1.pdf]

Article DOI: https://doi.org/10.3201/eid3009.240419

EID cannot ensure accessibility for supplementary materials supplied by authors. Readers who have difficulty accessing supplementary content should contact the authors for assistance.

Association of Intestinal Helminthiasis with Disseminated Leishmaniasis, Brazil

Study Area

The region of Corte de Pedra, situated in the southeast of the state of Bahia, Brazil, is composed of several rural municipalities spread over 8,000 km^2 with an estimated population of over 300,000 according to 2022 Brazilian census data (*1*). Bahia, a diverse state of national importance that contains intensely urbanized cities as well as vast agricultural lands, is the largest state by area and population in the northeast region of Brazil (*2*). This study was conducted at the Health Post of Corte de Pedra, which serves as the regional referral center for the diagnosis and treatment of CL and other forms of tegumentary leishmaniasis. The region lies between 49 and 1,640 feet above sea level and is part of the Atlantic rainforest biome, where substantial deforestation for the sake of large-scale agriculture of cacao, sugar cane, cassava, and banana has created opportunities for interaction between humans, *Lutzomyia* sandflies, and known non-human *Leishmania* reservoir species (*3*,*4*). The rural setting as well as data from previous studies suggest that coinfection by *L. braziliensis* and intestinal helminths would be common (*5*).

Patient Recruitment and Follow-up

Recruitment was conducted between January and December 2017 at the Health Post of Corte de Pedra in the endemic region of Corte da Pedra in Bahia, Brazil. Individuals between the ages of 5–70 with skin lesions that had been present for less than 60 days were eligible for enrollment. Patients were excluded if they presented with mucosal involvement of CL, were pregnant or breastfeeding, or had medical comorbidities such as diabetes mellitus, epilepsy, or immunodeficiency.

Criteria for diagnosis of CL were the presence of 1–9 well-demarcated cutaneous ulcers and detection by PCR of *L. braziliensis* DNA in a punch biopsy taken from a lesion. DL was defined as the presence of at least 10 cutaneous lesions located on at least two non-contiguous body parts and a positive PCR. At enrollment, participants had a leishmania skin test (Montenegro) placed and were asked to provide a stool sample. The presence of helminth infection was determined by rapid sedimentation, Baermann test, and the Kato-Katz method, while the number of helminth ova per gram of stool was quantified by Kato-Katz (*6*). All participants, regardless of enrollment status, were clinically evaluated and treated with 20 mg/kg/day of intravenous meglumine antimoniate for 20 days. One individual with DL was treated initially with amphotericin B due to the presence of over 1,500 skin lesions. Participants with a positive helminth examination were notified and treated in accordance with local guidelines.

Initial clinical examination consisted of an evaluation of the size and number of cutaneous lesions. On 60- and 90-day follow-up visits, participants were evaluated for the appearance of new lesions and response to treatment of existing lesions. Participants were considered cured by the presence of complete re-epithelialization without elevated borders of all CL or DL lesions within 90 days after the initiation of antimonial treatment. Participants that had not achieved cure at 90 days received an additional 20 mg/kg/day of intravenous meglumine antimoniate for 20–30 days. Five individuals with DL ultimately achieved cure after amphotericin B rescue therapy.

Laboratory Diagnosis

Once CL or DL were suspected based on the presence of characteristic skin lesions, diagnosis was confirmed by the presence of any detectable amount of amplification with quantitative real-time polymerase chain reaction (PCR) using primers specific to *L. braziliensis* kinetoplast DNA (kDNA) on punch biopsies collected from participants' cutaneous lesions upon initial clinical evaluation (*7*). Biopsies were performed on the elevated border of each participant's largest cutaneous lesion.

Additionally, participants were asked to supply a fecal sample for parasitological evaluation. For each sample, one slide was immediately prepared using both sedimentation and

Baermann techniques, and two slides were prepared using the Kato-Katz technique (*6*). Helminth burdens were calculated using the Kato-Katz method for *Ascaris lumbricoides*, *Necator americanus*, *Schistosoma mansoni*, *Taenia* spp., and *Trichuris trichiura* and were expressed as the number of helminth eggs per gram of stool.

Detection of *Leishmania braziliensis*

Leishmania braziliensis DNA was extracted from cutaneous tissue samples using the Maxwell Tissue DNA Kit (Promega Corporation, Madison, WI) and prepared for quantitative PCR as previously described (*8*). Definitive diagnosis of CL or DL was accomplished using custom DNA primers complementary to the kDNA3 region of the *L. braziliensis* genome (*7*).

Statistical Methods

Statistics were performed using the Python packages pandas and Matplotlib, using Mann-Whitney U test to compare the medians of numerical variables, chi-squared test for categorical variables, and linear regression analysis to compare dynamic associations between continuous variables (*9*,*10*). A P value <0.05 was considered to be a significant result. Survival analysis included all participants regardless of their ability to follow-up at 90 days and was presented as a Kaplan-Meier curve with statistical significance measured by log-rank test. The relationship between helminth burden and time to cure was analyzed using a multivariate adjusted Cox proportional-hazard model with participant age as a covariate. The hazard ratio was 0.48 (95% CI 0.29–1.59) for risk of cure in the DL group compared to the CL group.

Ethics Statement

The study was approved by the ethics committee of the Hospital Universitário Professor Edgard Santos, Salvador, Brazil (CAAE 01229212.0.0000.0049) and by the Institutional Review Board of Tulane University, New Orleans, LA. All participants provided written informed consent.

References

1. Instituto Brasileiro de Geografia e Estatística. Censo demográfico. 2022 [cited 2024 Aug 3]. https://censo2022.ibge.gov.br/panorama

2. Nitahara A. Estimativa da população do Brasil passa de 210 milhões, diz IBGE. 2010 [cited 2024 Jan 28]. https://agenciabrasil.ebc.com.br/economia/noticia/2019-08/estimativa-da-populacao-do-brasil-passa-de-210-milhoes-diz-ibge

3. Tabarelli M, Aguiar AV, Ribeiro MC, Metzger JP, Peres CA. Prospects for biodiversity conservation in the Atlantic Forest: lessons from aging human-modified landscapes. Biol Conserv. 2010;143:2328–40. https://doi.org/10.1016/j.biocon.2010.02.005

4. Gramiccia M, Gradoni L. The current status of zoonotic leishmaniases and approaches to disease control. Int J Parasitol. 2005;35:1169–80. PubMed https://doi.org/10.1016/j.ijpara.2005.07.001

5. O'Neal SE, Guimarães LH, Machado PR, Alcântara L, Morgan DJ, Passos S, et al. Influence of helminth infections on the clinical course of and immune response to *Leishmania braziliensis* cutaneous leishmaniasis. J Infect Dis. 2007;195:142–8. PubMed https://doi.org/10.1086/509808

6. World Health Organization. Basic laboratory methods in medical parasitology. Geneva: World Health Organization; 1991. p. 9–25 [cited 2024 Jan 28]. https://iris.who.int/handle/10665/40793

7. Weirather JL, Jeronimo SM, Gautam S, Sundar S, Kang M, Kurtz MA, et al. Serial quantitative PCR assay for detection, species discrimination, and quantification of *Leishmania* spp. in human samples. J Clin Microbiol. 2011;49:3892–904. PubMed https://doi.org/10.1128/JCM.r00764-11

8. Silva SC, Guimarães LH, Silva JA, Magalhães V, Medina L, Queiroz A, et al. Molecular epidemiology and in vitro evidence suggest that *Leishmania braziliensis* strain helps determine antimony response among American tegumenary leishmaniasis patients. Acta Trop. 2018;178:34–9. PubMed https://doi.org/10.1016/j.actatropica.2017.10.010

9. Hunter JD. Matplotlib: a 2D graphics environment. Computing in Science and Engineering. 2007;9:90–5 [cited 2024 Jan 28]. https://matplotlib.org/stable

10. McKinney W. Data structures for statistical computing in Python. In: Proceedings of the 9th Python in Science Conference, 2010 [cited 2024 Jan 28]. http://conference.scipy.org.s3-website-us-east-1.amazonaws.com/proceedings/scipy2010/mckinney.html

Appendix Table. Results of helminth examinations for individuals with cutaneous leishmaniasis and disseminated leishmaniasis.

Helminth species	Prevalence among individuals with cutaneous leishmaniasis, no. (%)	Prevalence among individuals with disseminated leishmaniasis, no. (%)	p value	Total prevalence among all individuals with leishmaniasis, no. (%)
Necator americanus	18.2 (18/99)	25 (5/20)	0.50	19.3 (23/119)
Trichuris trichiuris	18.2 (18/99)	5 (1/20)	0.14	16 (19/119)
Ascaris lumbricoides	12.1 (12/99)	10 (2/20)	0.79	11.8 (14/119)
Schistosoma mansoni	2 (2/99)	5 (1/20)	0.44	2.5 (3/119)
Strongyloides stercoralis	0 (0/99)	0 (0/20)	1	0 (0/119)
Total	40.4 (40/99)	40 (8/20)	0.97	40.3 (48/119)

Significance assessed by chi-squared test.
Parasitological evaluations were conducted using rapid sedimentation, Baermann, and Kato-Katz techniques from stool supplied on the date of first visit. The total helminth prevalence in each group is less than the sum of the prevalence of the individual helminth species, as some patients were coinfected with multiple helminth species.

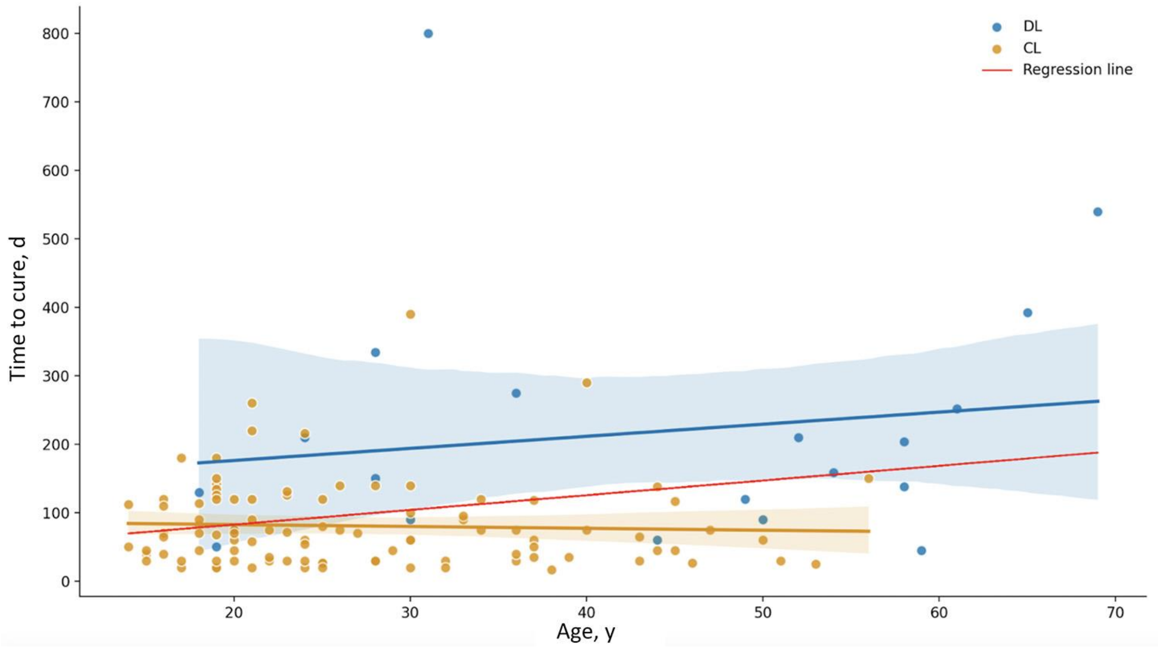

Appendix Figure 1. Linear regression analysis of participant age versus time to cure for individuals with cutaneous or disseminated leishmaniasis. For individuals with DL, there was no correlation between age and time to cure (R^2 0.06, p = 0.5). For individuals with CL, age is correlated with time to cure in CL but does not explain much of its variability (R^2 0.07, p = 0.04).

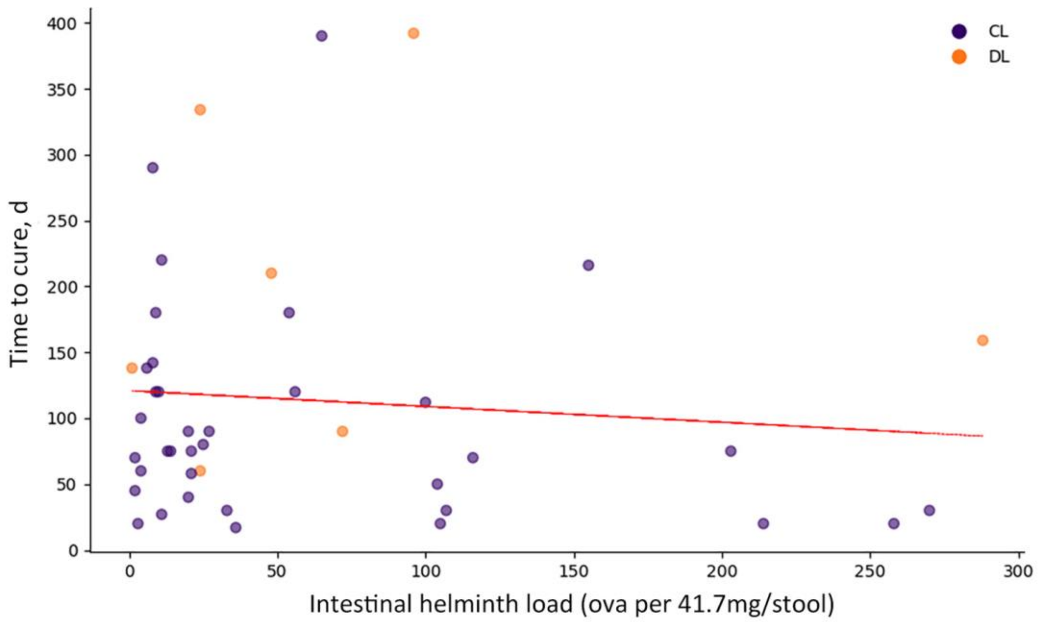

Appendix Figure 2. Linear regression analysis of participant intestinal helminth load versus time to cure for individuals with cutaneous or disseminated leishmaniasis. For individuals with CL and DL, there was no correlation between age and time to cure (R^2 0.04 and R2 0.00002, respectively). Participants with a negative coprological exam, an intestinal helminth load of >500 ova per 41.7 milligrams of stool, or a time to cure of >400 days were not included in the figure.
